# Synthesis and Polymerization of Thiophene‐Bearing 2‐Oxazolines and 2‐Oxazines

**DOI:** 10.1002/marc.202400946

**Published:** 2025-01-27

**Authors:** Aaron Lee, Julian Heck, Josef Goding, Robert Luxenhofer, Rylie A. Green

**Affiliations:** ^1^ Department of Bioengineering Imperial College London South Kensington London SW7 2AZ UK; ^2^ Department of Chemistry and Helsinki Institute of Sustainability Science Faculty of Science University of Helsinki Helsinki 00014 Finland

**Keywords:** cationic ring opening polymerization, crystallography, matrix‐assisted laser desorption/ionization mass spectrometry, polyoxazines, polyoxazolines

## Abstract

Intrinsically conductive polymers have garnered a great deal of attention for use in medical and bioelectronic applications. Despite this, challenges associated with the mechanical stability, processability, and fabrication of conducting polymers have limited their utility. To circumvent these limitations, thiophene substituted 2‐oxazolines (2Ox) and 2‐oxazines (2Ozi) are introduced, which can be polymerized to form a thermally stable and potentially melt‐processable polymers as precursors for conductive polymers. A series of such monomers are synthesized and yields above 50% are obtained for gram scale reactions. The monomers can subsequently be polymerized using standard cationic ring‐opening methods to yield thiophene‐bearing poly(2‐oxazoline)s (POx) and poly(2‐oxazine)s (POzi) with narrow to moderate dispersity. The polymers exhibit glass transition temperatures between 50 °C and 100 °C and thermal stability beyond 250 °C. Moreover, random copolymers can be produced by introducing aliphatic 2‐oxazolines during polymer synthesis, which facilitates tailoring of the polymer properties and may enable new applications in melt extrusion printing or electrospinning of precursors for conducting polymer systems. Overall, a facile approach is described for the synthesis of thiophene‐functionalized monomers and polymers, providing covalent integration of thiophenes that opens new avenues toward the generation of functional and stimuli‐responsive biomaterials.

## Graphical abstract

Thiophene‐bearing 2Ox and 2Ozi monomers are produced by utilizing the Witte and Seeliger method. The monomers are characterized by spectroscopic methods and x‐ray diffraction to resolve structural data and properties. Homopolymers synthesized using thiophene‐substituted monomers produced thermally stable products and glass transition temperature can be tuned by copolymerization.

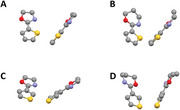

## 1 Introduction

2‐Oxazolines (2Ox) and 2‐oxazines (2Ozi) and their corresponding polymers have attracted a great deal of interest as synthetic building blocks for the generation of functional polymeric biomaterials.^[^ ^1^ ^]^ Synthetic versatility, biocompatibility, and processability have spurred investigation into their use for polymer‐drug conjugates, hemostatic patches, and as scaffolds for tissue engineering.^[^ ^2^, ^3^, ^4^, ^5^, ^6^, ^7^ ^]^ Poly(2‐oxazolines) (POx) and poly(2‐oxazines) (POzi) can be prepared by living cationic ring‐opening polymerization (LCROP), allowing tuneability and application‐specific engineering of physical and chemical properties.^[^ ^8^, ^9^ ^]^ Side group modification allows fine‐tuning of critical parameters such as hydrophilicity and hydrophobicity,^[^ ^10^ ^]^ thereby tuning of thermoresponsive behavior and influencing hydrogelation for specific biomaterial applications.^[^ ^11^, ^12^ ^]^ In addition to highly definable chemistry with narrow dispersity, POx and POzi can be processed using advanced manufacturing techniques such as electrospinning, melt electrowriting, and fused deposition modeling.^[^ ^4^, ^5^, ^6^, ^13^, ^14^, ^15^ ^]^

Unlike inert materials, stimuli‐responsive and functional biomaterials that can actively interact with cells and tissues offer the ability to influence their behavior.^[^ ^16^, ^17^, ^18^ ^]^ Electroactive polymers such as poly(3,4‐ethylenedioxythiophene):poly(styrene sulfonate) (PEDOT:PSS) exhibit both electronic and ionic conduction and allow a virtually seamless transition between traditional silicon‐based computers to soft and wet biological circuitry.^[^ ^19^, ^20^, ^21^ ^]^ While conducting polymers such as PEDOT:PSS have demonstrated excellent performance as a charge transfer interface and have the ability to be modified for improved biological activity, they are critically hampered by poor mechanical stability and limited processability.^[^ ^22^, ^23^, ^24^ ^]^ Methods to impart electroactivity to traditionally inert polymer systems include blending and coating.^[^ ^25^, ^26^ ^]^ However, to be able to fully address the many challenges and applications in which conductive biomaterials could be used to improve health and patient outcomes, new hybrid polymers that can be shaped into complex architectures while retaining electrical conductivity are needed.

Achieving functional and processable polymers is of great interest for smart tissue interfaces. Recent work to improve the processability of conductive polymer systems has resulted in the development of macromonomers that incorporate electroactive segments.^[^ ^27^, ^28^ ^]^ These systems have demonstrated the potential for melt electrowriting or electrospinning to form nanoscale and microscale fibre architectures suitable for engineering conductive cell scaffolds.^[^ ^28^ ^]^ However, material conductivity remains poor due to the use of short conductive segments and hindered inter‐chain charge transfer due to steric effects associated with macromonomer design. An alternate approach to achieving high intra‐chain charge mobility and thermal or electrically‐driven processing is to assemble a co‐chain structure, through which a processable polymer backbone and a conducting polymer backbone are covalently bonded and run in parallel as a ladder‐type structure.^[^ ^29^ ^]^ In this way, charge mobility along the conducting polymer chain can be preserved alongside processability for functional cell‐supportive biomedical applications.

Herein, novel 2Ox and 2Ozi featuring thiophene side chains are introduced.^[^ ^30^ ^]^ We will demonstrate that these molecules can be polymerised using standard cationic ring opening polymerisation approaches and that the polymers can be dissolved in common solvents for solution‐based processing. In addition, their thermal properties make them amenable for thermal processing. These polymers add to the available toolbox to generate functional electroactive polymers that can be easily modified for specific applications and address unmet challenges, particularly in biomaterials and neural interfacing. By integrating thiophene‐functionality onto 2Ox and 2Ozi monomers, the resultant polymers have the potential to sustain a covalently formed structural network alongside a conducting polymer network.

## 2 Results and Discussion

**Scheme 1:**
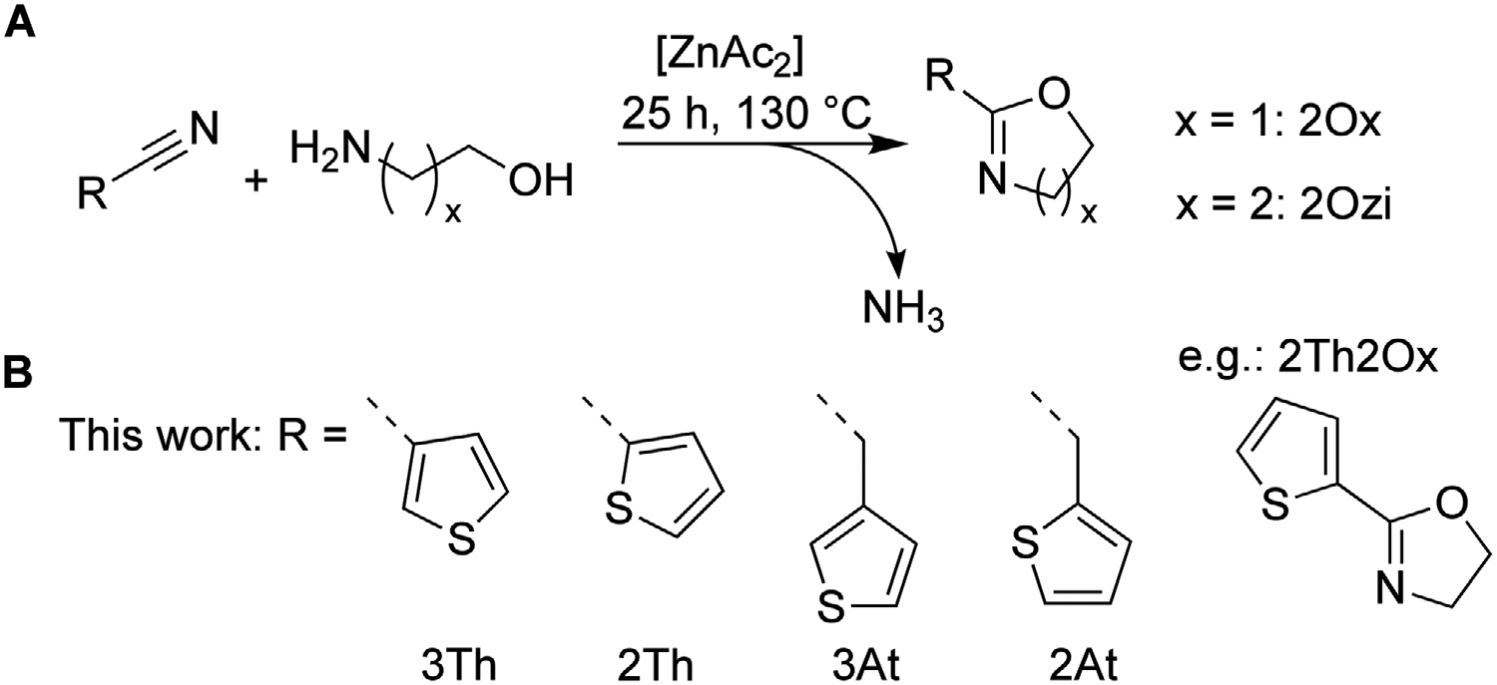
A) Schematic overview of 2‐R‐2‐oxazolines and 2‐R‐2‐oxazines synthesis from nitrile‐bearing thiophenes and either ethanolamine (x = 1) or propanolamine (x = 2), respectively and B) overview of monomers introduced in this work along with the abbreviations of the different moieties (see also Table 1).

**Figure 1:**
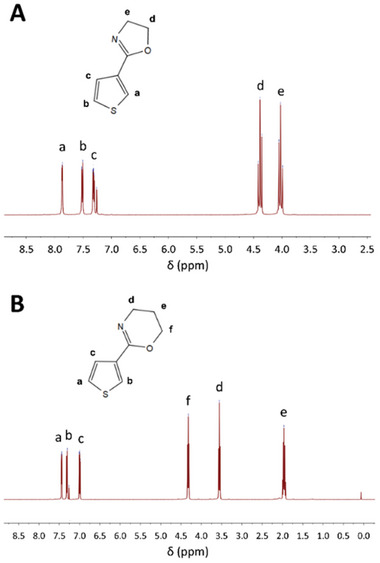
Example of ^1^H‐NMR spectra (300 MHz, CDCl_3_) and signal assignment for selected monomers. A) 2‐(thiophen‐3′‐yl)‐2‐oxazoline (3Th2Ox) and B) 2‐(thiophen‐3′‐yl)‐2‐oxazine (3Th2Ozi).

Thiophene‐based 2Ox and 2Ozi monomers were synthesized based on the method described by Witte and Seeliger (**Scheme** **1**).^[^ ^30^ ^]^ Briefly, a nitrile‐bearing thiophene was reacted with an amino alcohol in equimolar quantities in the presence of zinc acetate as a catalyst while refluxed at 130 °C. The synthesis was performed as a one pot reaction for ≈25 h, as shorter reaction times did not result in full conversion, while longer reaction times led to side reactions that decreased the overall yield of purified product (**Table** **1**). All monomers showed good solubility in diethyl ether and insolubility in water. Reaction products were isolated by diluting the cooled mixture with water and extracting the organic phase into diethyl ether before further purification by distillation. Monomers were subsequently characterized by NMR spectroscopy (**Figure** **1**; Figures S1‐S6, Supporting Information). Crystallization of thiophene‐derivatized molecules from toluene was performed for X‐ray diffraction and subsequent structure determination.

**Table 1:** Summary of monomers synthesized and general properties (abbreviation, yield, state at room temperature (RT), melting point, and boiling point).

Product	Abbreviation	Yield [%]	State [RT]	MP [°C]	BP [°C at 0.01 mbar]
2‐(thiophen‐2′‐yl)‐2‐oxazoline	2Th2Ox	74.8	Solid	23‐30	102 – 107
2‐(thiophen‐3′‐yl)‐2‐oxazoline	3Th2Ox	92.4	Solid	27 – 29	105 – 115
2‐(acetothiophen‐2′‐yl)‐2‐oxazoline	2At2Ox	53.2	Liquid	–	80 – 90
2‐(acetothiophen‐3′‐yl)‐2‐oxazoline	3At2Ox	64.0	Liquid	–	80 – 85
2‐(thiophen‐2′‐yl)‐2‐oxazine	2Th2Ozi	77.3	Solid	19 – 25	110 – 120
2‐(thiophen‐3′‐yl)‐2‐oxazine	3Th2Ozi	89.5	Solid	24 – 28	110 – 120
2‐(acetothiophen‐2′‐yl)‐2‐oxazine	2At2Ozi	53.2	Liquid	–	85 – 95
2‐(acetothiophen‐3′‐yl)‐2‐oxazine	3At2Ozi	60.2	Liquid	–	85 – 95

Mostly moderate to high yields (70‐90%) were obtained for 2‐(thiophen‐2′‐yl)‐2‐oxazoline (2Th2Ox) and 2‐(thiophen‐2′‐yl)‐2‐oxazine (2Th2Ozi), which are solids at room temperature. Somewhat lower yields of 50–60% were achieved for 2‐(acetothioph‐2′‐yl)‐2‐oxazoline (2At2Ox) and 2‐(acetothiophen‐2′‐yl)‐2‐oxazine (2At2Ozi), which are liquid at room temperature.

A previous example of a synthesis to produce a thienyl‐substituted oxazoline involved using a carboxylic acid precursor to a similar yield of 70%, though no subsequent polymerisation was undertaken.^[^ ^31^ ^]^ The observed lower yields when using acetonitrile thiophenes in comparison to carbonitrile thiophenes are attributed to a lowered reactivity due to the additional methylene group which acts as an electron donating group and consequently increases the electron density of the nitrile group.^[^ ^32^ ^]^ Improved reaction yields of ≈10% were observed for β‐carbon thiophene derivatives. Differences in the reactivity of substituted thiophenes have been described for thiophene carboxylic acids with thiophene‐2‐carboxylic acid indicating higher reactivity arising from an internal conformer as supported by computational methods.^[^ ^33^ ^]^ As the linear nitrile group does not possess other conformations, the difference in reactivity is likely to arise from the electronic density distribution around the ring relative to the nitrile moiety for each regioisomer.^[^ ^34^, ^35^, ^36^ ^]^

Spectroscopic analysis was performed for the purified compounds by ^1^H‐NMR. Representatively, for 3Th2Ox, peaks between 4.0 and 4.4 ppm can be attributed to the protons adjacent to the N and O in the 2Ox rings while the peaks between 7.3 and 7.9 ppm are attributed to the thiophene ring with a residual solvent peak associated with CDCl_3_ present at 7.26 ppm (Figure 1A; Sections S1–S6, Supporting Information). Similarly, thiophene signals in 3Th2Ozi are located between 7.0 and 7.5 ppm. Due to the additional methylene group in the 2Ozi ring, the protons bound to the C‐4 and C‐6 position appear at lower chemical shifts (4.32 and 3.56 ppm), respectively (Figure 1B). Additionally, a signal attributed to the protons at the 5‐carbon in the 2Oz appears at 1.96 ppm. The additional carbon in the ring has been attributed with lower ring tension and reduced polymerization rates of 2Ozi compared to 2Ox.^[^ ^37^ ^]^ However, when the two types are copolymerized, 2Ozi can preferentially polymerize over 2Ox.^[^ ^38^ ^]^

**Figure 2:**
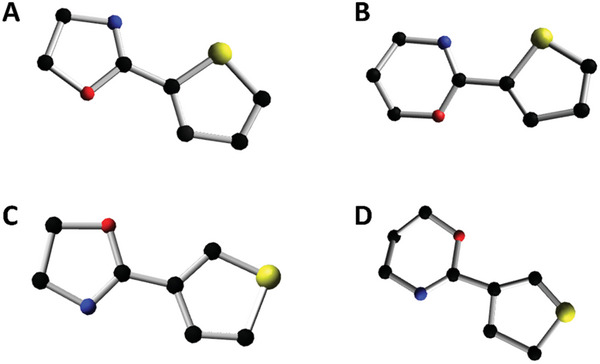
Resolved structures of thiophene‐derivatives obtained by X‐ray crystallography of purified products prepared from toluene with carbon atoms shown in black, nitrogen atoms shown in blue, oxygen atoms shown in red and sulphur atoms shown in yellow. The following molecules are shown: A) 2‐(thiophen‐2′‐yl)‐2‐oxazoline, B) 2‐(thiophen‐2′‐yl)‐2‐oxazine, C) 2‐(thiophen‐3′‐yl)‐2‐oxazoline and D) 2‐(thiophen‐3′‐yl)‐2‐oxazine.

Crystallographic data was obtained for the 2Ox and 2Ozi monomers as they are solid at room temperature and readily formed single crystals from toluene (**Figure** **2**; Figure S7, Tables S1 and S2, Supporting Information). Formation of the crystalline phase is supported by the presence of the planar thiophene systems.^[^ ^39^ ^]^ The five‐member 2Ox ring results in a fully planar molecule, in comparison the additional carbon in oxazine ring is deflected out of plane which is associated with lowered ring tension.^[^ ^38^ ^]^ For all crystals, a disorder of 180° along the bonding axis of the thiophene ring was observed for 2Ox and 2Oz, respectively. Crystal structures for 2Ox and 2Ozi monomers are rather rare, as most monomers are liquids or crystallize poorly. However, recently the crystal structure for 2‐benzhydryl‐2‐oxazine as well as a cubane oxazoline were reported.^[^ ^40^, ^41^ ^]^ As there is free rotation between the two ring systems, no preferred orientation of the thiophene ring is expected.

The highest yields of ≈90% we obtained for 2‐(thiophen‐3′‐yl)‐2‐oxazoline (3Th2Ox) and 2‐(thiophen‐3′‐yl)‐2‐oxazine (3Th2Ozi). For our intended later use to obtain conjugated polymers using the pending thiophene moieties, both 3Th2Ox and 3Th2Ozi have the advantage of preserving two α‐carbon positions in the thiophene ring that are necessary for a potential secondary polymerization and therefore, the potential to form π‐conjugated systems, essential for establishing electrical conductivity.

**Figure 3:**
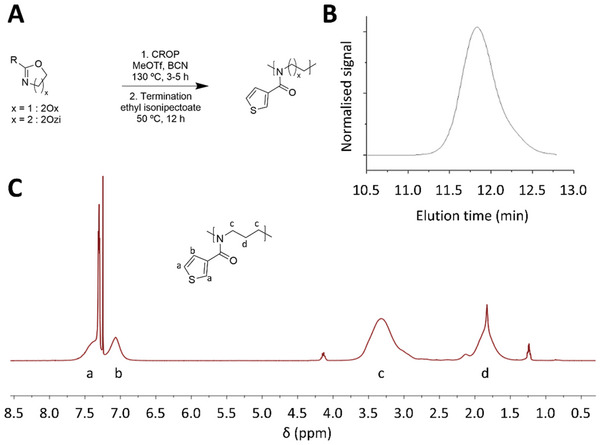
a) General polymerization scheme used for synthesis of poly(oxazolines/oxazines) via CROP and initiated with MeOTf in benzonitrile (BCn), b) Representative GPC of poly(2(thiophen‐3′‐yl)‐2‐oxazine) (P3Th2Ozi_25_) showing monomodal molecular weight distribution, c) ^1^H‐NMR spectrum of P(3Th2Ozi)_25_ (CDCl_3_, 298 K, 300 MHz) with signal assignment.

Polymerization of 3Th2Ox and 3Th2Ozi was achieved by cationic ring‐opening co‐polymerization (CROP) in benzonitrile at 130 °C (**Figure** **3**a). Methyl trifluoromethanesulfonate (MeOTf) was used as initiator while ethyl isonipectotate was used for functional termination of the polymer chains. The monomer to initiator ratio was varied between 25:1 and 50:1 with the polymerization time kept to ≈5 h for higher degree of polymerization (*n* = 50). The various thiophene‐POx and POzi could be polymerized to near 100% conversion and were obtained as solids. P3Th2Ox was characterized by gel permeation chromatography (GPC) with a dispersity of 1.14 (Figure 3b). NMR spectroscopy shows peak broadening of alkyl protons 1.8 ppm and 3.2 ppm (Figure 3c). The remaining synthesised POx and POzi were characterised by NMR and GPC with a dispersity between 1.09 and 1.47 (Table S3, Supporting Information). Poly(2‐(thiophen‐2′‐yl)‐2‐oxazoline) (P2Th2Ox) and poly(2‐(thiophen‐3′‐yl)‐2‐oxazoline) (P3Th2Ox) were insoluble in chloroform and were characterized using GPC and MALDI‐TOF MS (Figure S9, Supporting Information). By comparison, poly(2‐(thiophen‐3′‐yl)‐2‐oxazine) (P3Th2Ozi) possessed improved solubility with a low dispersity with Đ = 1.09 – 1.27 and obtainable as a pure powder. Polymerization of 2At2Ox, 3At2Ox, 2At2Ozi, and 3At2Ozi gave lower yields ranging from 50 – 60% and lower molecular weights (Table S3, Supporting Information).

**Figure 4:**
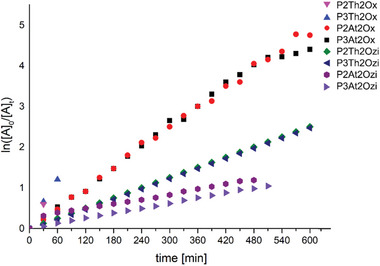
First order kinetics of polymerisation of thiophene‐based 2‐oxazolines and 2‐oxazines. Linearity preserved for the first six hours of the reaction.

Polymerization of thiophene oxaz(ol)ines using CROP displayed typical kinetic characteristics of living polymerization (**Figure** **4**). Higher rates of polymerization were observed for the smaller 2Ox heterocycle than for the six‐membered 2Ozi counterpart. No major rate differences were observed for 2‐carbon or 3‐carbon derivatives, with acetothiophene derivatives having slower kinetics than carbonitrile thiophene derivatives. Trends in kinetics follow expected behavior with larger, bulkier pendant groups associated with slower reaction rates.^[^ ^10^ ^]^

**Figure 5:**
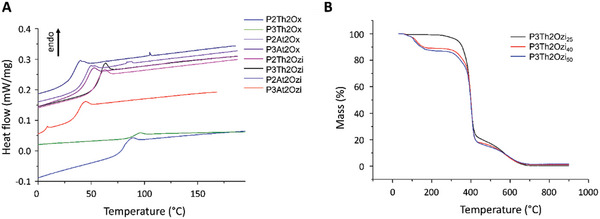
Thermal characterization of polymer behavior. A) differential scanning calorimetry from the second cycle. B) thermogravimetric analysis of poly(2‐(thiophen‐3′‐yl)‐oxazine) with different degrees of polymerisation (*n* = 25, 40, 50).

The thermal properties of the polymers were assessed by thermogravimetric analysis and differential scanning calorimetry (DSC) (**Figure** **5**). DSC was performed over three cycles from ‐50 – 250 °C. Data obtained for homopolymers with a degree of polymerisation of 25 and 50 show clear glass transitions (T_g_) between 40 °C and 100 °C (Figure 5A; Table S3, Supporting Information). Interestingly, T_g_ values appear to be unaffected by the doubling of the degree of polymerization (DP), which is somewhat unexpected for such short DP. It has also been shown that for certain POx that the T_g_ may be molecular mass independent and is strongly influenced by bulky pendant groups, such as the thiophene heterocycle.^[^ ^38^, ^39^ ^]^ Thermogravimetric analysis of P3Th2Ozi with different DP show that lower molecular weight polymers have minimal mass change at temperatures exceeding 200 °C (Figure 5B). Mass loss at higher molecular weights and lower temperature (≈100 °C) during heating was attributed to residual solvent loss from the polymer with most thermal degradation occurring at temperatures ≈400 °C. Drying of lower molecular weight polymers was more effective at removing solvent with no initial mass loss present. Notably, high boiling point solvents are known to be difficult to remove and it has been shown that polyoxazolines can adsorb moisture from atmosphere.^[^ ^42^ ^]^

Solubility of polymers was determined in common solvents as a route to solution‐based processing applications (Table S4, Supporting Information). The synthesized monomers exhibited good solubility in most common solvents such as chloroform, acetonitrile (ACN), and hexafluoroisopropanol (HFIP). Of the homopolymers, POx exhibited the broadest solubility in these solvents, a temperature‐dependent solubility in alcohols, and swelling behavior in water. As thiophene‐based POx and POzi are largely insoluble in water, purification must take place by precipitation or dialysis into an organic solvent.

To improve polymer processability and compatibility with 3D‐printing techniques such as fused deposition modeling and melt electrowriting, a lower glass transition (T_g_) is preferred. P3Th2Ozi possess a T_g_ ≈100 °C, while the additional carbon linking the thiophene ring to the backbone in P3At2Ozi resulted in increased mobility and a significantly decreased T_g_ to ≈40 °C (Table S3, Supporting Information). The synthetic versatility of thiophene‐substituted 2Ox and 2Ozi was further investigated by exploring the effect of copolymerization to adjust properties. Co‐polymerization with 2‐*n*‐propyl‐2‐oxazine (nPrOzi) was chosen due to the lower T_g_ (9 °C) of PnPrOzi, which should significantly lower the T_g_ of copolymers.^[^ ^11^, ^43^ ^]^ Indeed, DSC analysis reveals single T_g_ ranging from 38 to 51 °C for the copolymers P(3Th2Ozi‐co‐nPrOzi), down from 59 °C for P3Th2Ozi, suggesting the absence of phase separation between 3Th2Ozi and nPrOzi. The versatility of this approach demonstrates that polymer properties can be controlled and engineered as desired according to the needs of processing requirements. The various monomers and polymers could be promising as precursors for functional electroactive polymers, which hold immense promise for engineering of 3D structured conductive tissue scaffolds as well as patternable bioelectronics. To date, many of the leading conducting polymer candidates suffer from friability and poor processability which have made them unsuitable for applications requiring complex geometries. Consequently, a new family of melt and solution processable monomers and polymers based on thiophene‐modified heterocyclic compounds as presented here could help to overcome these barriers. Thiophenes are a particularly attractive option as a pendant group due to thermal stability and ease of synthesis and open avenues to integration with conducting polymer segments.^[^ ^44^ ^]^ To produce a processable conducting polymer, ongoing work explores the ability to produce ladder‐style polymer systems by polymerizing the pending thiophenes moieties. As an alternative, POx/POzi chains could be used to bridge adjacent polythiophene molecules to confer additional mechanical stability or as handles for subsequent chemical modification. In this work, polymerization of the 2Ox/2Ozi through ring‐opening was performed first. Given that the polymerization mechanisms of POx and POzi are orthogonal with the oxidative or electrochemical polymerization of thiophene derivatives, it may be possible to first establish a conjugated backbone using the thiophene followed by CROP. It is important to note that the electronic performance of polythiophenes depends on the accessibility of dopants and counterions.^[^ ^45^ ^]^ Moreover, charge transfer in aqueous solutions through ionic conduction is known to be important for the excellent performance of conducting polymer electrodes in bioelectronics for recording and stimulation.^[^ ^46^, ^47^ ^]^ Ensuring that both electronic and ionic charge transport can occur unimpeded in these dual polymer systems will be critical for bridging the gap between easily processable biomaterials and functional conducting polymers.

## 3 Conclusion

Thiophene‐bearing 2Ox and 2Ozi monomers were produced by utilizing the Witte and Seeliger method. The monomers were characterized by spectroscopic methods and X‐ray diffraction to resolve structural data and properties. These monomers retained the ability of 2Ox and 2Ozi monomers to be polymerized via cationic ring opening polymerization and produced polymers with low to moderate dispersity. While homopolymers synthesized using thiophene‐substituted monomers produced thermally stable products, the glass transition temperature can be tuned through a combination of monomer selection and copolymerization. Our approach allows for one‐step preparation of thiophene‐derivatized monomers at a gram scale and can be further tailored to bring electroactive properties to a diverse chemical family of processable and stimuli‐responsive biomaterials.

## 4 Experimental Section

### Materials

All commercially available chemicals were purchased by Sigma Aldrich or Acros Organics and used without further purification unless otherwise described. Toluene was dried for 72 h over molecular sieves (4 Å). 2‐Ethyl‐2‐oxazoline, benzonitrile, Methyl‐2‐2oxazoline were dried by refluxing over CaH_2_ and distilled afterwards prior use. FeCl_3_ was dried under vacuum at 50 °C for 24 h prior use. If not stated different, all reactions and work‐up procedures were performed under inert conditions (water‐free Argon atmosphere using a CaCl_2_ filter). For inert conditions during synthesis, all reactions and workup were performed using either a standard Schlenk System or a glovebox (LabMaster 130, Mbrazn, Garching, Germany) under inert atmosphere of dry, oxygen‐free argon. The water impurity of the glovebox was detected and had an average of less than 5 ppm.

### Monomer Synthesis

Synthesis of all monomers was carried out analogously to the synthesis route as described by Witte and Seeliger. As all monomers are fully new to literature, cleaning, and purification procedures were adapted accordingly. Briefly, 1 eq of monomer was mixed with 1 eq of alkanoamine and 0.25 eq zinc acetate dihydrate in a 100 mL round flask with refluxing condenser and stirred at 130 °C under argon atmosphere for 25 h. The mixture was cooled down and approx. 100 mL of water was added. The mixture was extracted three times by using ethyl ether (3×50 mL). The organic phases were collected and washed two times with water and dried over MgSO_4_. After removing the organic solvent, a raw product was obtained. For further purification either sublimation distillation or distillation was performed.

### Polymer Synthesis

In a two‐neck flask, the monomers were dissolved in dry benzonitrile under inert conditions. Methyl triflate was added as initiator and stirred at 130 °C for 3–5 h for polymerization. The reaction mixture was cooled to 50 °C, ethyl isonipecotate was added for termination and stirring continued at room temperature for at least 12 h. For purification, the cooled reaction mixture was precipitated in at least four times the amount of cold diethyl ether and then centrifuged. The solvent was decanted and the precipitated polymer was dissolved in a maximum of 25 mL chloroform. The reaction mixture was then precipitated again in four times the amount of ice‐cold ether. This purification step was repeated at least four times. As a final step, the polymer obtained was dried for at least 72 h under vacuum at room temperature. The synthesis presented within was confirmed using at least 5 replicates for each synthesis strategy and a minimum of 10 repetitions for the key products.

### NMR Spectroscopy

Spectra were acquired using a Bruker Fourier 300 (^1^H 300.12 MHz, ^13^C: 75.47 MHz) and a Bruker Fourier 400 equipped with a Bruker BioSpin (Rheinstetten, Germany) at 298 K. Spectra were calibrated to residual protonated solvent signals (CDCl_3_ 7.26 ppm, HDO 4.67 ppm, D_6_‐DMS 2.50 ppm). Products were dissolved in the appropriate solvent at a concentration of 15 mg mL^−1^. Successful reactions were determined by analyzing the integral of peaks that corresponded to the theoretical expected values of protons at defined chemical shifts. Multiplicities of signals are denoted as s, singlet; d, dublet; q, quartet; quin, quintet; dt, doublet of triplets; m, multiplet; b, broad; ps, pseudo. Coupling constants were calculated by Mestrenova (version 6.0.2‐5475). NMR assignments are supported by additional 2D experiments.

### X‐Ray Diffraction

X‐ray diffraction was performed on a Bruker D8 Advance and a Stoe IPDS with a voltage and current of 30 kV and 53.3 mA, respectively. A Cu‐K or Mo‐X alpha radiation source was used. X‐ray intensity was determined for 2θ between 10° and 80° in 0.015° angle steps with 0.7 s dwell time and a rotation speed of 15 rpm. Samples were mounted on a zero‐background holder single crystal silicon. Measurements of the background were performed using a beam knofe and DIFFRAC:EVA. SHELXL‐2018 and SIR‐2004 were used to determine heavy atom positions and remaining non‐hydrogens were located from successive difference Fourier map calculations.

### Mass Spectrometry

Mass spectrometry was performed with an Agilent Technologies 6200 series TOF/6500 series Q‐TOF B.08.00. Ionisation was achieved by electron spray and peaks recorded as mass‐to‐charge ratios versus counts.

### Gel Permeation Chromatography

Molecular weights determined by GPC were determined in a Polymer Standard Service Security (PSS, Mainz, Germany) system (pump mod. 1260 infinity, MDS RI detector mod. 1260 infinity (Agilent Technologies, Santa Clara, California, USA), precolumn PSS GRAM 10 µm (50×8 mm), PSS GRAM 30 Å 10 µm (300×8 mm), and PSS GRAM 1000 Å 10 µm (300×8 mm)) at 313 K. DMF was supplemented with 1 g L^−1^ lithium bromide and the flow rate was adjusted to 1 mL min^−1^. Prior to each measurement, the samples were filtered through a 0.2 µm Teflon filter (Thermo Scientific, Schwerte, Germany) to remove particles. The system was calibrated against PEG standards with molar masses ranging from 106 g mol^−1^ to 100 kg mol^−1^. Data were processed using WinGPC Unichrom V.8.20 Build 5350 software.

For polymers dissolved in hexafluoroisopropanol the Polymer Standard Service Security (PSS, Mainz, Germany) system (pump mod. 1260 infinity, MDS RI detector mod. 1260 infinity (Agilent Technologies, Santa Clara, California, USA), precolumn PSS PFG linear M 7 µm (50×8 mm), PSS PFG linear M; 2 columns: 7 µm (300×8 mm)) at 313 K was used. HFIP was supplemented with 3 g L^−1^ potassium triflate and the flow rate was adjusted to 0.7 mL min^−1^. Calibration was performed using PEG standards with molar masses ranging from 200 g mol^−1^ to 100 kg mol^−1^.

### Differential Scanning Calorimetry

Differential scanning calorimetry was performed on a DSC 204F1 Phoenix (NETZSCH, Selb, Germany) under nitrogen atmosphere (20.0 mL min^−1^). Samples were placed in aluminium pans with crimped lids and thermally cycled between ‐50 °C and 200 °C with a temperature ramp speed of 10 K min^−1^.

### Thermogravimetric Analysis

Thermogravimetric analysis of the polymers was performed on a TG 209F1 IRIS (NETZSCH, Selb, Germany). The samples (5 – 10 mg) were added to aluminium oxide crucibles (NETZSCH, Selb, Germany) and heated under nitrogen from 50 to 850 °C with a heating rate of 10 K min^−1^ while detecting the mass loss. The corresponding NETZSCH Proteus – Thermal Analysis – V.5.2.1 software was used to evaluate the obtained spectra and OriginLab 9.0 was used for plotting.

## Conflict of Interest

The authors declare no conflict of interest.

## Supporting information



Supporting Information

## Data Availability

The data that support the findings of this study are available from the corresponding author upon reasonable request.
