# Supplementary material for: Synthesis and Polymerization of Thiophene‐Bearing 2‐Oxazolines and 2‐Oxazines
Source: Macromol Rapid Commun. 2025 Jan 27;46(6):2400946. doi: 10.1002/marc.202400946 (PMC11925325; doi:10.1002/marc.202400946)
Supplement: Supplementary file 1 — Supporting Information [file MARC-46-2400946-s001.docx]

# Supporting Information

## Synthesis of 2-(thiophen-2’yl)-2-oxazoline (2Th2Ox)

2-thiophenecarbonitrile (5.00 g, 0.05 mol, 1eq) was reacted with ethanolamine (2.80 g, 0.05 mol, 1eq) in the presence of zinc acetate dihydrate (0.25 g, 1.15 mmol, 0.025 eq). Reaction produced a colourless solid identified as 2-(thiophen-2’-yl)-2-oxazoline.

Yield: 5.24 g, 0.03 mol, 74.8 % (colourless solid)

^1^H-NMR (300 MHz, CDCl_3_, δ ppm): 7.59‒7.58 (d, J = 3.5 Hz, 1H), 7.45‒7.43 d, J = 5.0 Hz, 1H), 7.09‒7.05 (m, 1H), 4.46‒4.39 (t, J = 9.5 Hz, 2H), 4.07‒4.01 (t, J = 9.3 Hz, 2H)

^13^C-NMR (75 MHz, CDCl_3_, δ ppm): 160.40 (N=**C**‒O), 130.40 (N/O‒C‒α**C**‒thiophene) 130.14(α**C**‒thiophene), 129.71(β**C**‒thiophene), 127.57 (β**C**‒thiophene), 68.05 (O‒**C**‒CH_2_), 55.02 (N‒**C**‒CH_2_)

MS (ESI) m/Z: [M]^+^ 154.07, bp: 102 ‒ 107 °C (0.01 mbar)

## Synthesis of 2-(thiophen-3’-yl)-oxazoline) (3Th2Ox)

Ethanolamine (5.60 g, 0.09 mol, 1eq) was reacted with 3-thiophencarbonitrile (10.0 g, 0.09 mol, 1eq) in the presence of zinc acetate dihydrate (0.50 g, 2.29 mmol, 0.025 eq). Reaction produced a colourless solid identified as 2-(thiophen-3’-yl)-2-oxazoline.

Yield: 12.5 g 0.08 mol, 89.5 % (colourless solid)

^1^H-NMR (300 MHz, CDCl_3_, δ ppm): 7.80 (d, J = 2.6 Hz, 1H), 7.45 (d, J = 5.1 Hz, 1H), 7.25 (dd, J = 4.9, 3.0 Hz, 1H), 4.33 (t, J = 9.4 Hz, 3H), 3.96 (t, J = 9.4 Hz, 3H)

^13^C-NMR (75 MHz, CDCl_3_, δ ppm): 163.43 (N=**C**‒O), 131.47 (β**C**‒thiophene) 128.59(α**C**‒thiophene), 127.32(α**C**‒thiophene), 126.99 (β**C**‒thiophene), 69.09 (O‒**C**‒CH_2_), 59.28 (N‒**C**‒CH_2_)

MS (ESI) m/Z: [M]^+^ 154.05, bp: 105 ‒ 115 °C (0.01mbar)

## Synthesis of 2-(acetothiophen-2’-yl)-2-oxazoline (2At2Ox)

Ethanolamine (2.48 g, 0.04 mol, 1eq) was reacted with 2-thiophenacetonitrile (5.00 g, 0.04 mol, 1eq) in the presence of zinc acetate dihydrate (0.22 g, 1.15 mmol, 0.025 eq).  Reaction produced a light-yellow liquid identified as 2-(acetothiophen-2’-yl)-2-oxazoline.

Yield: 8.85 g, 0.05 mol, 60.2% (light yellow liquid)

^1^H-NMR (300 MHz, CDCl_3_, δ ppm): 7.13 (m, 1H), 6.96‒6.94 (m, 2H), 4.31‒4.24 (t, J = 9.5 Hz, 2H), 3.89‒3.83 (m, 4H)

^13^C-NMR (75 MHz, CDCl_3_, δ ppm): 166.03 (N=**C**‒O), 159.40 (α**C**‒C‒oxazoline), 126.86 (α**C**‒thiophene) 126.57(β**C**‒thiophene), 124.80(β**C**‒thiophene), 67.87 (O‒**C**‒CH_2_), 54.54 (N‒**C**‒CH_2_), 29.00 (thiophene‒**C**‒oxazoline)

MS (ESI) m/Z: [M]^+^: 168.04, bp: 80 ‒ 85 °C (0.01 mbar)

## Synthesis of 2-(acetothiophen-3’-yl)-2-oxazoline (3At2Ox)

3-thiophenacetonitrile (10.0 g, 0.08 mol, 1eq) was reacted with ethanolamine (4.96 g, 0.08 mol, 1eq) in the presence of zinc acetate dihydrate (0.44 g, 2.03 mmol, 0.025 eq). Reaction produced a colourless liquid identified as 3-(acetothiophen-3’-yl)-2-oxazoline.

Yield: 8.68 g, 0.05 mol, 64.0 % (colourless liquid)

^1^H-NMR (300 MHz, CDCl_3_, δ ppm): 7.29‒7.26 (dd, J = 7.7, 2.9 Hz, 1H), 7.16‒7.15 (m, 1H), 7.06‒7.05 (d, J = 4.9 Hz, 1H), 4.30‒4.23 (t, J = 9.5 Hz, 1H), 3.89‒3.82 (t, J = 9.4 Hz, 1H), 3.65 (s, 2H)

^13^C-NMR (75 MHz, CDCl_3_, δ ppm): 166.56 (N=**C**‒O), 134.87 (α**C**‒thiophene), 128.42 (α**C**‒thiophene) 125.75(β**C**‒C‒oxazoline), 122.35(β**C**‒thiophene), 67.67 (O‒**C**‒CH_2_), 54.52 (N‒**C**‒CH_2_), 29.36 (thiophene‒**C**‒oxazoline)

MS (ESI) m/Z: [M]^+^ 168.06 , bp: 80 ‒ 90 °C (0.01 mbar) 

## Synthesis of 2-(thiophen-2’-yl)-2-oxazine) (2Th2Ozi)

2-thiophencarbonitrile (4.00 g, 0.04 mol, 1eq) was reacted with 3-amino-1-propanol (2.75 g, 0.04 mol, 1eq) in the presence of zinc acetate dihydrate (0.20 g, 0.91 mmol, 0.025 eq). Reaction produced a colourless solid identified as 2-(thiophen-2’-yl)-2-oxazine.

Yield: 5.16 g, 0.03 mol, 77.3 % (colourless solid)

^1^H-NMR (300 MHz, CDCl_3_, δ ppm): 7.48‒7.47 (d, J = 2.5 Hz, 1H), 7.35‒7.32 (m, 1H), 7.04‒7.00 (m, 1H), 4.36‒4.34 (dd, J = 8.9, 5.3 Hz, 2H), 3.59‒3.57 (dd, J = 9.1, 5.8 Hz, 2H), 2.00‒1.98 (m, 2H)

^13^C-NMR (75 MHz, CDCl_3_, δ ppm): 152.64 (N=**C**‒O), 138.38 (N/O‒C‒α**C**‒thiophene) 128.13(α**C**‒thiophene), 127.24(β**C**‒thiophene), 128.01 (β**C**‒thiophene), 65.41 (O‒**C**‒CH_2_), 42.50 (N‒**C**‒CH_2_), 21.95 (H_2_C‒**C**‒CH_2_),

MS (ESI) m/Z: [M]^+^ 168.05, bp: 110 ‒ 120 °C (0.01mbar)

## Synthesis of 2-(thiophen-3’-yl)-2-oxazine) (3Th2Ozi)

2-thiophenecarbonitrile (25.0 g, 0.23 mol, 1eq) was mixed with 3-amino-1-propanol (17.2 g, 0.23 mol, 1eq) and zinc acetate dihydrate (1.26 g, 5.73 mmol, 0.025 eq) in a 100 ml round flask with a refluxing condenser and stirred at 130°C under argon atmosphere for 25 hours. The mixture was cooled down and extracted three times with 50 mL ethyl ether. The organic phase was collected, washed with water twice and dried over MgSO_3_. While removing the solvent, a crude darkish red product was obtained. Purification by distillation/sublimation and resolving in dry toluene resulted in a colourless solid (35.3 g, 0.21 mol, 92.4%).

Yield: 35.3 g, 0.21 mol, 92.4 % (colourless solid)

^1^H-NMR (300 MHz, CDCl_3_, δ ppm): 7.45‒7.44 (d, J = 3.0 Hz, 1H), 7.32‒7.30 (d, J = 5.1 Hz 1H), 7.01‒7.98 (m, 1H), 4.32 (t, J = 5.3 Hz 2H), 3.56 (t, J = 5.9 Hz 2H), 1.96 (m, 2H)

^13^C-NMR (75 MHz, CDCl_3_, δ ppm): 153.12 (N=**C**‒O), 137.06 (N/O‒C‒β**C**‒thiophene) 126.61(α**C**‒thiophene), 126.27 (α**C**‒thiophene), 125.35 (β**C**‒thiophene), 65.12 (O‒**C**‒CH_2_), 42.41 (N‒**C**‒CH_2_), 21.95 (H_2_C‒**C**‒CH_2_),

MS (ESI) m/Z: [M]^+^ 168.04, bp: 110 ‒ 120 °C (0.01mbar)

## Synthesis of 2-(acetothiophen-2’-yl)-2-oxazine (2At2Ozi)

2-thiophenacetonitrile (5,00 g, 0,04 mol, 1eq) was reacted with 3-amino-1-propanol (3,05 g, 0,04 mol, 1eq) in the presence of zinc acetate dihydrate (0,22 g, 1,02 mmol, 0,025 eq).  Reaction produced a yellow-orange liquid identified as 2-(acetothiophen-2’-yl)-2-oxazine.

Yield: 3.91 g, 0.02 mol, 53.2 % (yellow-orange liquid)

^1^H-NMR (300 MHz, CDCl_3_, δ ppm): 7.18‒7.16 (dd, J = 5.0, 1.4 Hz, 1H), 6.95‒6.90 (m, 2H), 4.19‒4.16 (t, J = 5.5 Hz, 2H), 3.65 (s, 2H), 3.42‒3.38 (t, J = 5.9 Hz, 2H), 1.90‒1.83 (m, 2H)

^13^C-NMR (75 MHz, CDCl_3_, δ ppm): 158.14 (N=**C**‒O), 138.50 (α**C**‒C‒oxazine), 126.69 (α**C**‒thiophene) 125.94(β**C**‒thiophene), 124.35(β**C**‒thiophene), 65.21 (O‒**C**‒CH_2_), 42.36 (N‒**C**‒CH_2_), 36.81 (thiophene‒**C**‒oxazine), 21.62 (NC‒**C**‒CO)

MS (ESI) m/Z: [M]^+^ 182.06, bp: 85 ‒ 95 °C (0.01mbar)

## Synthesis of 2-(acetothiophen-3’-yl)-2-oxazine (3At2Ozi)

3-thiophenacetonitrile (10.0 g, 0.08 mol, 1eq) was reacted with 3-amino-1-propanol (6.10 g, 0.08 mol, 1eq) in the presence of zinc acetate dihydrate (0.44 g, 2.03 mmol, 0.025 eq). Reaction produced a colourless liquid identified as 2-(acetothiophen-3’-yl)-2-oxazine.

Yield: 8.85 g, 0.05 mol, 60.2 % (colourless liquid)

^1^H-NMR (300 MHz, CDCl_3_, δ ppm): 7.18‒7.16 (dd, J = 4.9, 3.0 Hz, 1H), 6.95‒6.91 (m, 2H), 4.18‒4.15 (t, J = 5.5 Hz, 2H), 3.64 (s, 2H), 3.41‒3.37 (t, J = 5.8 Hz, 2H), 1.89‒1.82 (m, 2H)

^13^C-NMR (75 MHz, CDCl_3_, δ ppm): 162.33 (N=**C**‒O), 142.70 (α**C**‒thiophene), 138.38 (α**C**‒thiophene) 126.03(β**C**‒C‒oxazine), 124.42(β**C**‒thiophene), 66.77 (O‒**C**‒CH_2_), 44.44 (N‒**C**‒CH_2_), 38.79 (thiophene‒**C**‒oxazine), 19.65 (NC‒**C**‒CO)

MS (ESI) m/Z: [M]^+^ 182.08, bp: 85 ‒ 95 °C (0.01 mbar)

## Poly(2-(thiophen-3’-yl)-2-oxazine) (P2Th3Ozi)

According to the general procedure, P2Th3Ozi was synthesised by using 2-thiophene-3-oxazine (15.0 g, 0.09mol, 25eq) in 45 mL benzonitrile Methyl triflate (0.59 g, 3,59 mmol, 1eq) and ethyl isonipecotate (1.69 g, 10.8 mmol, 3eq).

GPC (HFIP): M_n_ =  2.9 kg/mol, Đ = 1.09

^1^H-NMR (300 MHz, CDCl_3_, δ ppm): 7.31 (2H), 7.06 (1H), 3.33 (4H), 1.83 (2H)

Poly(2-(thiophen-3’-yl)-2-oxazoline) (P2Th3Ox)

According to the general procedure, P2Th3Ox was synthesised by using 2-thiophene-3-oxazoline (10.0 g, 0.06 mol, 25 eq) in 30 mL benzonitrile Methyl triflate (0.43 g, 2.61 mmol, 1 eq) and ethyl isonipecotate (1.23 g, 7.84 mmol, 3 eq). The polymerisation time was stopped after three hours due to the insolubility of the polymer in benzonitrile. According to this, the ^1^H-NMR was measured with additional hexafluoroisopropanol (HFIP).

GPC (HFIP): M_n_ =  1.5 kg/mol, Đ = 1.14

^1^H-NMR (300 MHz, CDCl_3_, δ ppm): 7.33 (2H), 7.07 (1H), 3.60 (4H)

Poly(2-(thiophen-2’-yl)-2-oxazine) (P2Th2Ozi)

According to the general procedure, P2T2Ozi was synthesised by using 2-thiophene-2-oxazine (15.0 g, 0.09 mol, 25 eq) in 45 mL benzonitrile Methyl triflate (0.59 g, 3.59 mmol, 1 eq) and ethyl isonipecotate (1.69 g, 10.8 mmol, 3 eq).

GPC (HFIP): M_n_ =  7.5 kg/mol, Đ = 1.27

^1^H-NMR (300 MHz, CDCl_3_, δ ppm): 7.40 (1H), 7.27 (1H), 7.00 (1H), 3.49 (4H), 1.99 (2H)

Poly(2-(thiophen-2’-yl)-2-oxazoline) (P2Th2Ox)

According to the general procedure, P2Th2Ox was synthesised by using 2-thiophene-2-oxazoline (10.0 g, 0.06 mol, 25 eq) in 30 mL benzonitrile Methyl triflate (0.43 g, 2.61 mmol, 1 eq) and ethyl isonipecotate (1.23 g, 7.84 mmol, 3 eq). The ^1^H-NMR was measured with additional HFIP due to the insolubility analogous to P2Th2Ox.

GPC (HFIP): M_n_ =  1.1 kg/mol, Đ = 1.19

^1^H-NMR (300 MHz, CDCl_3_, δ ppm): 7.40 (1H), 7.27 (1H), 7.00 (1H), 3.49 (4H), 1.99 (2H)

Poly(2-(acetothiophen-3’-yl)-2-oxazoline) (P2At3Ox)

According to the general procedure, P2At3OX was synthesised by using 2-thiophene-3-oxazoline (10.0 g, 0.06 mol, 25 eq) in 30 mL benzonitrile Methyl triflate (0.39 g, 2.39 mmol, 1 eq) and ethyl isonipecotate (1.13 g, 7.18 mmol, 3 eq).

GPC (HFIP): M_n_ =  3.1 kg/mol, Đ = 1.12

^1^H-NMR (300 MHz, CDCl_3_, δ ppm): 7.27 (1H), 7.23 (1H), 7.05 (1H), 3.71 (2H), 3.35 (4H)

Poly(2-(acetothiophen-3’-yl)-2-oxazine) (P2At3Ozi)

According to the general procedure, P2At3Ozi was synthesised by using 2-thiophene-3-oxazine (10.0 g, 0.06 mol, 25 eq) in 30 mL benzonitrile Methyl triflate (0.36 g, 2.21 mmol, 1 eq) and ethyl isonipecotate (1.04 g, 6.63 mmol, 3 eq).

GPC (HFIP): M_n_ =  5.9 kg/mol, Đ = 1.36

^1^H-NMR (300 MHz, CDCl_3_, δ ppm): 7.27 (1H), 7.04 (1H), 6.97 (1H), 3.64 (2H), 3.23 (4H), 1,65 (2H)

## Poly(2-(acetothiophen-2’-yl)-2-oxazine) (P2At2Ozi)

According to the general procedure, P2At2Ozi was synthesised by using 2-thiophene-2-oxazine (10.0 g, 0.06 mol, 25 eq) in 30 mL benzonitrile Methyl triflate (0.36 g, 2.21 mmol, 1 eq) and ethyl isonipecotate (1.04 g, 6.63 mmol, 3 eq).

GPC (HFIP): M_n_ =  3.1 kg/mol, Đ = 1.39

^1^H-NMR (300 MHz, CDCl_3_, δ ppm): 7.16 (1H), 6.91‒6.86 (2H), 3.81 (2H), 3.28 (4H), 1.73 (2H)

## Synthesis of Poly(2-(acetothiophen-2’-yl)-2-oxazoline) (P2At2Ox)

According to the general procedure, P2At2Ox was synthesised by using 2-thiophene-2-oxazoline (10.0 g, 0.06 mol, 25 eq) in 30 mL benzonitrile Methyl triflate (0.39 g, 2.39 mmol, 1 eq) and ethyl isonipecotate (1.13 g, 7.18 mmol, 3 eq).

GPC (HFIP): M_n_ =  5.8 kg/mol, Đ = 1.47

^1^H-NMR (300 MHz, CDCl_3_, δ ppm): 7.14 (1H), 6.89 (2H), 3.91 (2H) 3.41 (4H)

## NMR spectra


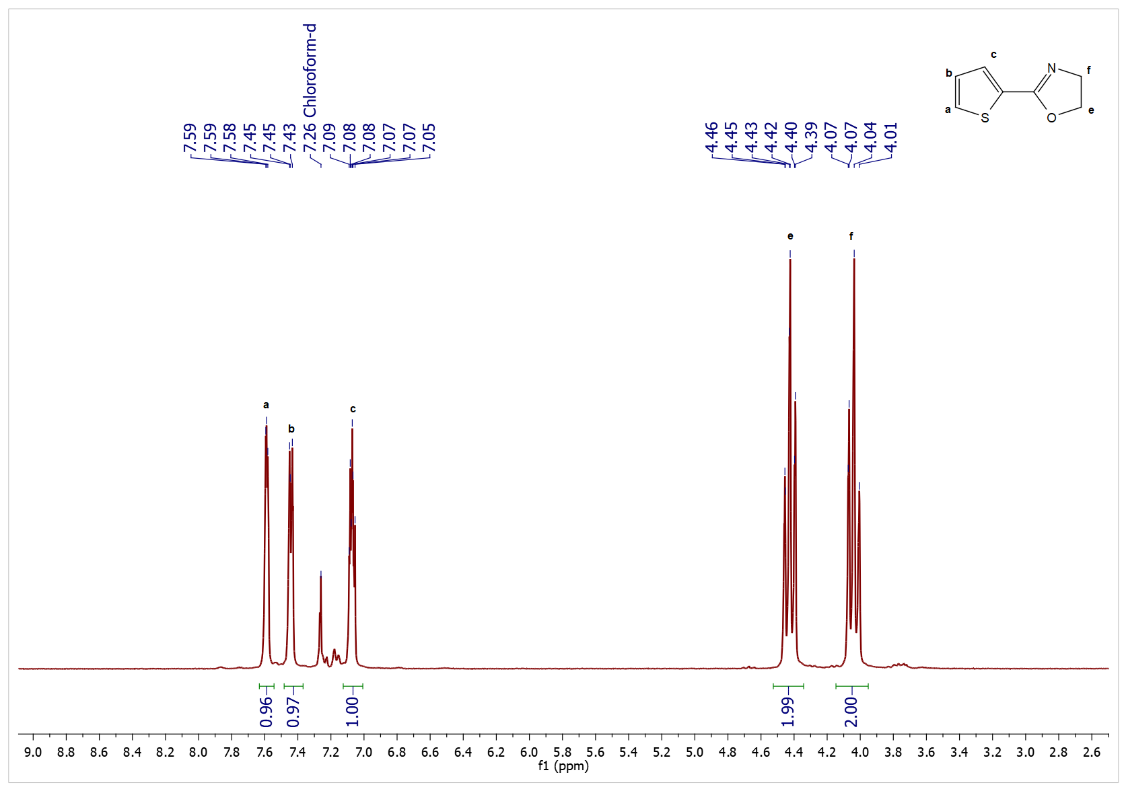


**Supporting Information Figure 1.** ^1^H-NMR spectra of 2-(thiophen-2’-yl)-2-oxazoline (2Th2Ox) with assigned peaks (CDCl_3_, 300 MHz).


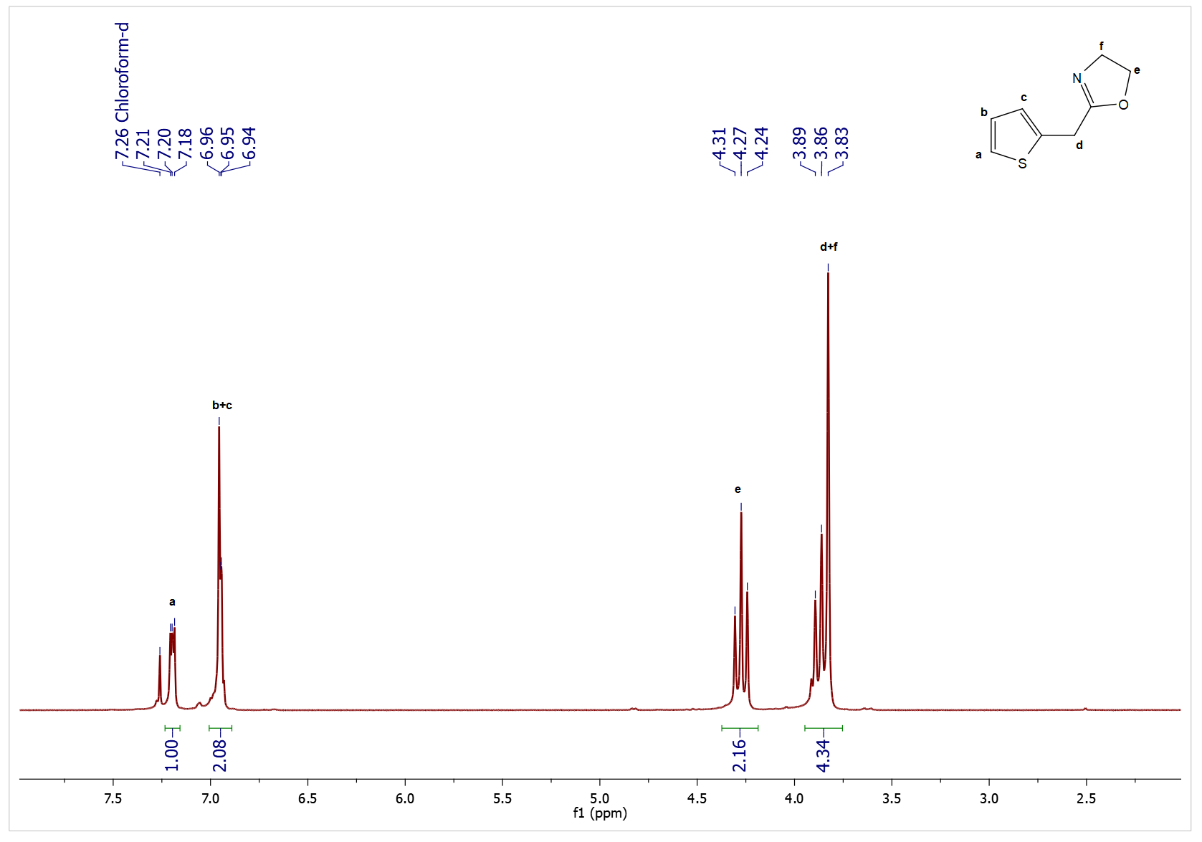


**Supporting Information Figure 2.** ^1^H-NMR spectra of 2-(acetothiophen-2’yl)-2-oxazoline (2At2Ox) with assigned peaks (CDCl_3_, 300 MHz).


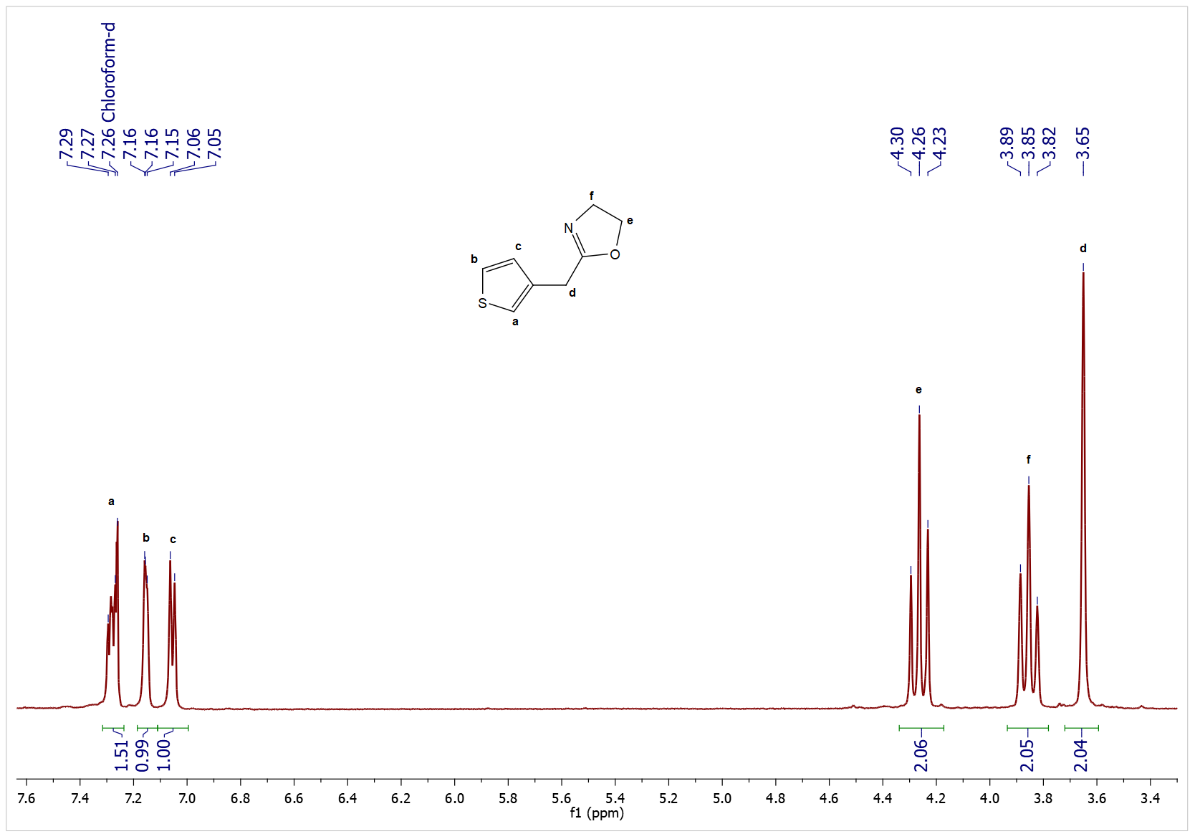


**Supporting Information Figure 3.** ^1^H-NMR spectra of 2-(acetothiophen-3’-yl)-2-oxazoline (3At2Ox) with assigned peaks (CDCl_3_, 300 MHz).


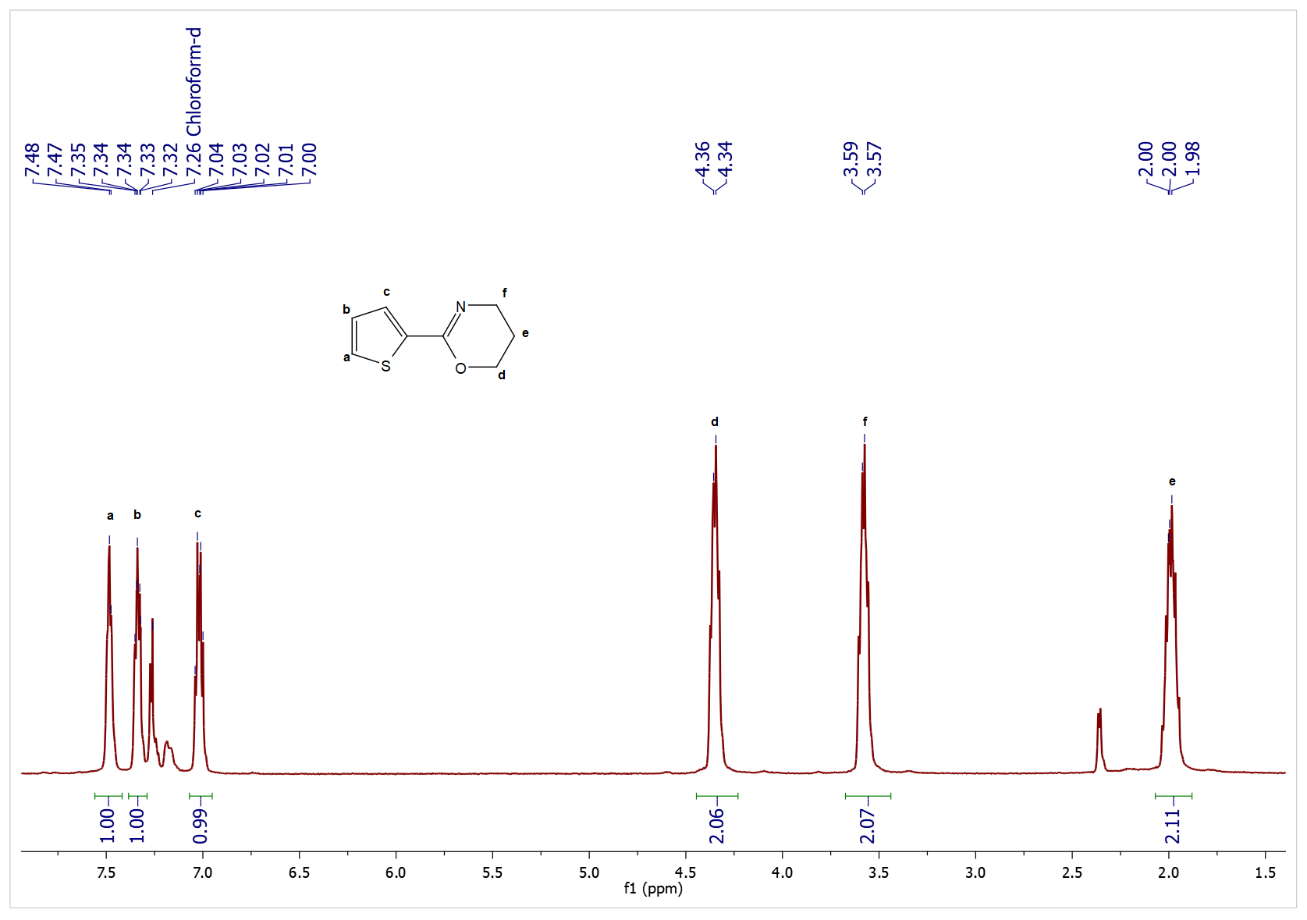


**Supporting Information Figure 4.** ^1^H-NMR spectra of 2-(thiophen-2’-yl)-2-oxazine (2Th2Ozi) with assigned peaks (CDCl_3_, 300 MHz).


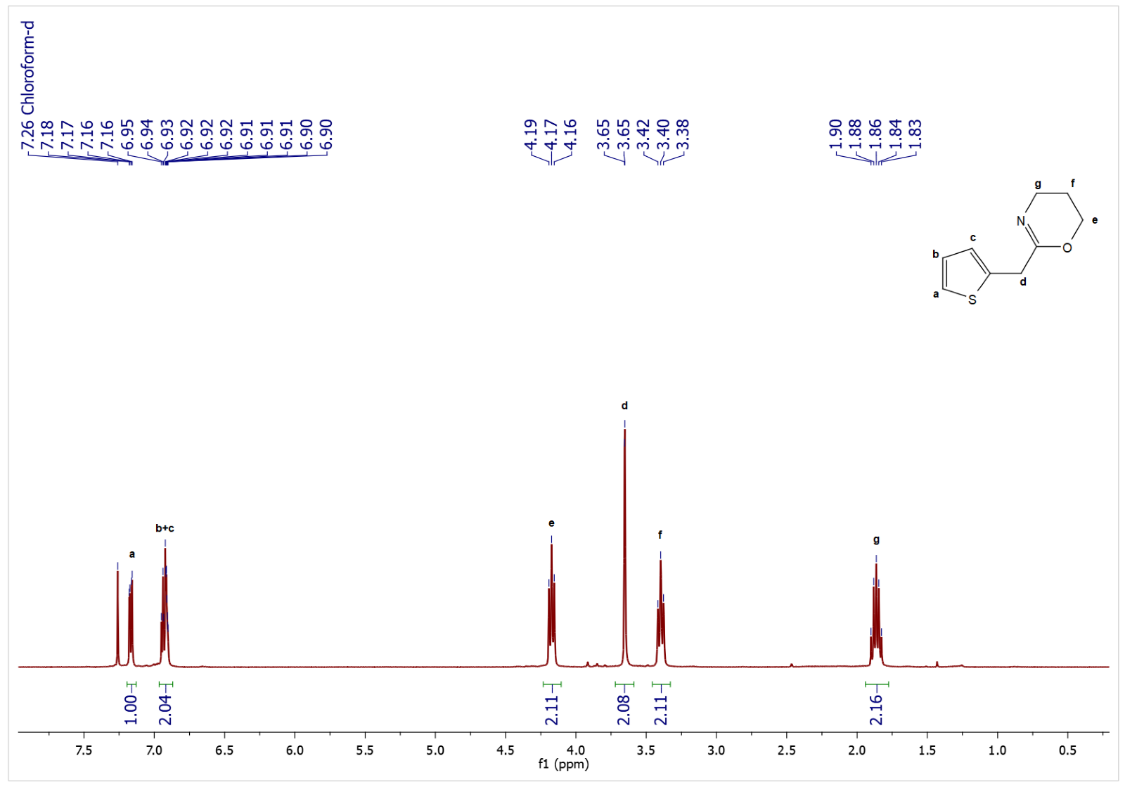


**Supporting Information Figure 5.** ^1^H-NMR spectra of 2-(acetothiophen-2’-yl)-2-oxazine (2Th2Ozi) with assigned peaks (CDCl_3_, 300 MHz).


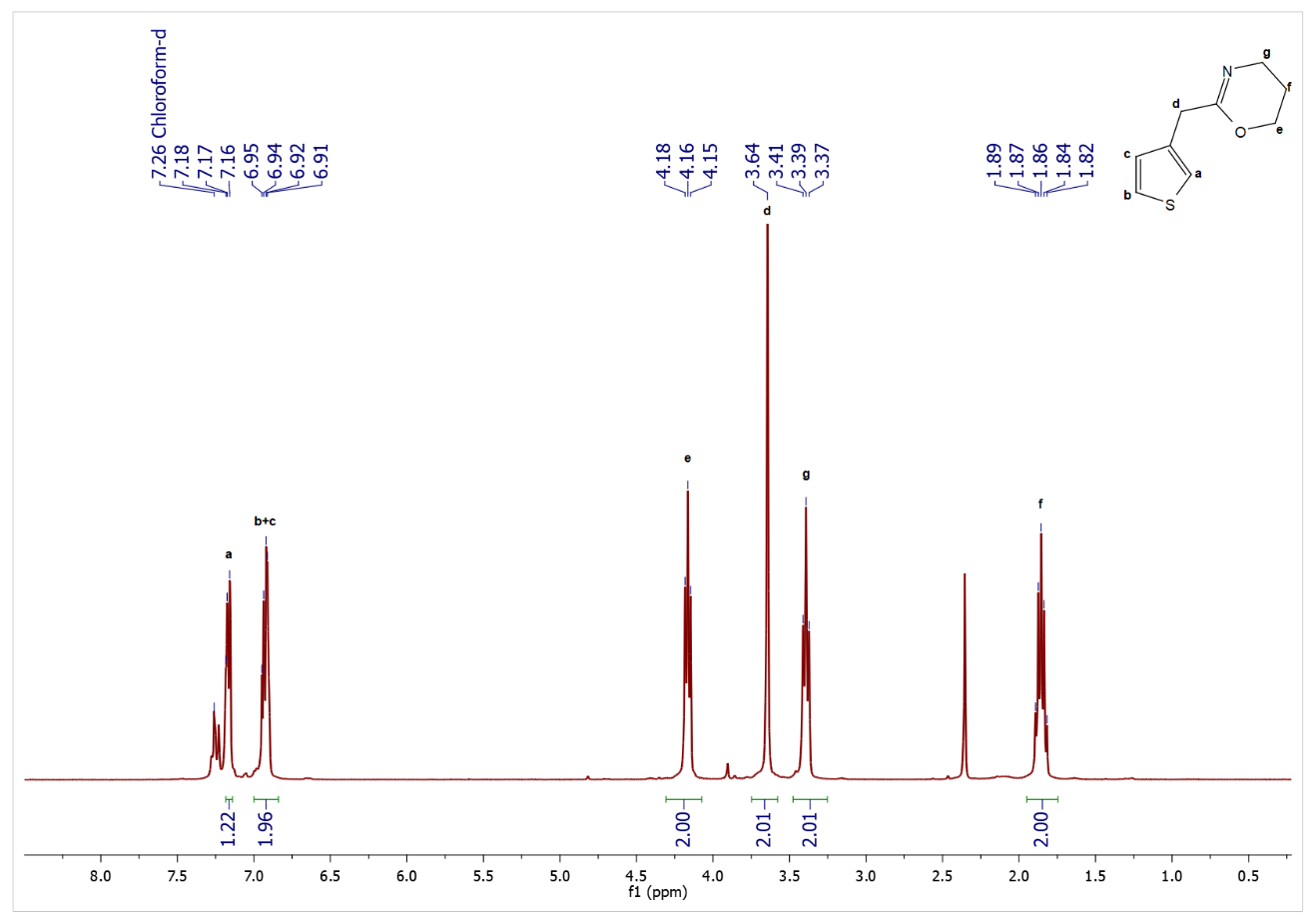


**Supporting Information Figure 6.** ^1^H-NMR spectra of 2-(acetothiophen-3’-yl)-2-oxazine (3Th2Ozi) with assigned peaks (CDCl_3_, 300 MHz).

## X-ray crystallographic data for structure determination


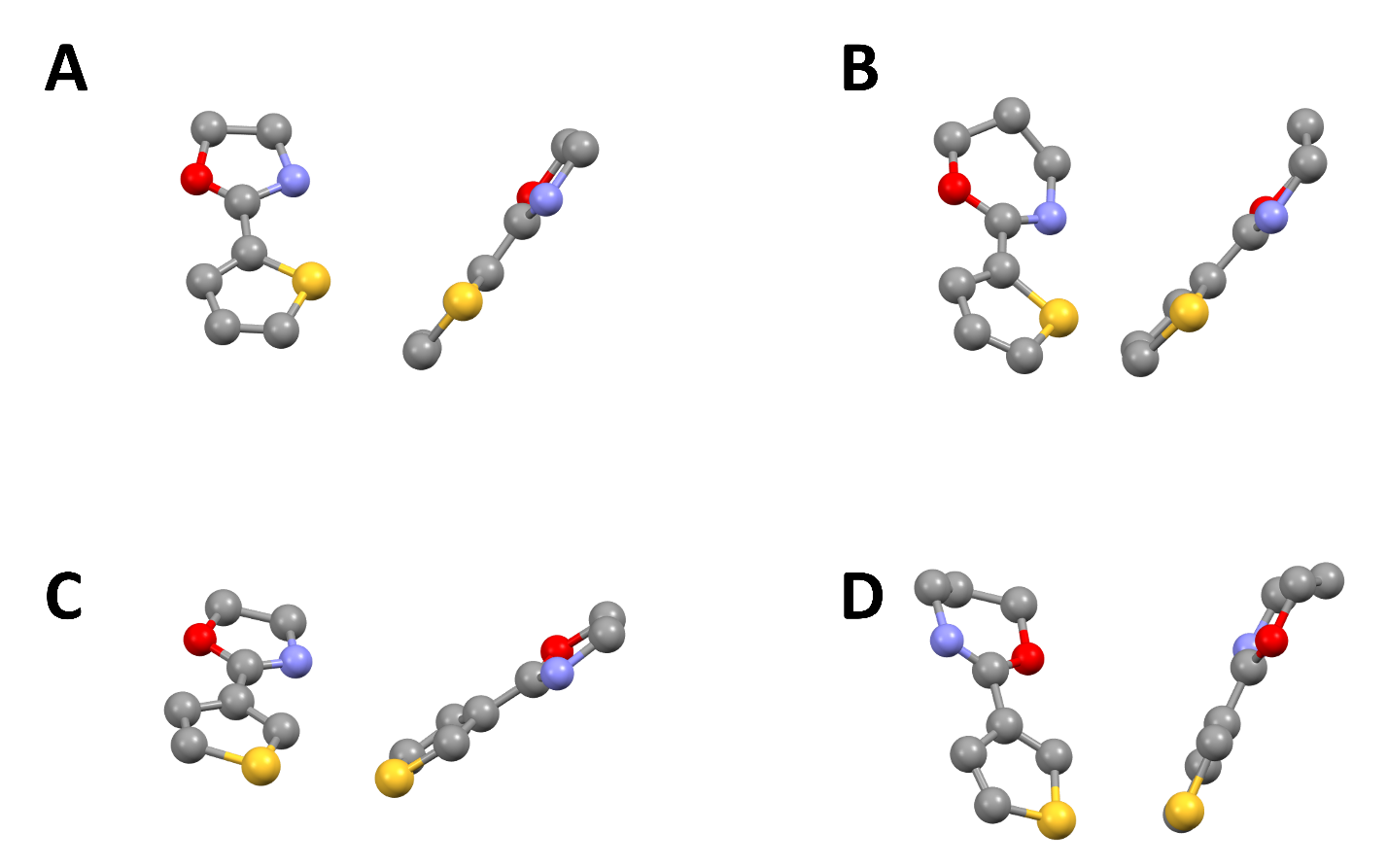


**Supporting Information Figure 7.** Additional perspectives of resolved structures obtained from x-ray diffraction with carbon atoms shown in grey, nitrogen atoms shown in blue, oxygen atoms shown in red and sulphur atoms shown in yellow. The following molecules are described: A) 2-(thiophen-2’-yl)-2-oxazoline, B) 2-(thiophen-2’-yl)-2-ozazine, C) 2-(thiophen-3’-yl)-2-oxazoline, D) 2-(thiophen-3’-yl)-2-oxazine.

**Supporting Information table 1.**  Summary of key bond distances and angles determined by x-ray crystallography of solid monomers.

|  | 2Th2Ox | 3Th2Ox | 2Th2Ozi | 3Th2Ozi |
| --- | --- | --- | --- | --- |
| Bond distance (Å) |  |  |  |  |
| S – C | 1.66 – 1.67 | 1.67 – 1.76 | 1.71 – 1.74 | 1.68 – 1.71 |
| O – C | 1.36 | 1.33 | 1.32 | 1.36 |
| N = C | 1.27 | 1.29 | 1.27 | 1.27 |
| Bond angle (°) |  |  |  |  |
| C – S – C | 94.5 | 90.7 | 90.7 | 91.9 |
| N = C – O | 119.6 | 118.2 | 128.1 | 128.1 |

**Supporting Information table 2.** Selected crystallographic data for 2Th2Ox, 3Th2Ox, 2Th2Ozi, 3Th2Ozi obtained by x-ray diffraction.

|  | **2Th2Ox** | **3Th2Ox** | **2Th2Ozi** | **3Th2Ozi** |
| --- | --- | --- | --- | --- |
| **Formula** | C_7_H­_7­_NOS | C_7_H­_7­_NOS | C_8_H­_9­_NOS | C_8_H­_9­_NOS |
| **Molecular weight** | 153.2 g/mol | 153.2 g/mol | 167.22 | 167.22 |
| **Crystal system** | orthorhombic | monoclinic | monoclinic | Monoclinic |
| **Space group** | Pnma | P 2_1_/c | P 2_1_ /n | P 2_1_/c |
| **a (Å)** | 10.7303(13) | 10.7476(12) | 7.8486(8) | 9.5031(3) |
| **b (Å)** | 10.1694(14) | 6.5050(5) | 10.8107(9) | 8.0553(2) |
| **c (Å)** | 6.6720(8) | 11.1934(12) | 9.6243(10) | 20.8306(6) |
| **α (°)** | 90 | 90 | 90 | 90 |
| **β (°)** | 90 | 114.329(8) | 100.190(8) | 91.427(3) |
| **γ (°)** | 90 | 90 | 90 | 90 |
| **V (Å^3^)** | 7.28.06(16) | 713.07(13) | 803.81 | 1594.11(8) |
| **Crystal size (mm³)** | 0.422 x 0.278 x 0.187 | 0.040 x 0.370 x 0.880 | 0.090 x 0.470 x 0.750 | 0.397 x 0.320 x 0.230 |
| **Z** | 4 | 4 | 4 | 8 |
| **Temp. (K)** | 173 | 193 | 193 | 173 |
| **dcalc (gcm^-1^)** | 1.398 | 1.427 | 1.382 | 1.394 |
| **Abs. coeff. μ (mm^-1^)** | 3.341 | 0.375 | 0.339 | 3.097 |
| **Reflections Collected** | 1932 | 4072 | 4251 | 5296 |

## Molecular weight characterisation of P2Th2Ox and P3Th2Ox by MALDI-TOF

Polymers P2Th2Ox and P3Th2Ox were analysed by matrix-assisted laser desorption/ionisation-time of flight mass spectrometry (MALDI-TOF) due to insolubility. Molar mass agrees with the expected theoretical value for degree of polymerisation and follows a Poisson distribution. These characteristics are typical for living polymerisation reactions. Due to the undefined spectra, a more precise molar mass distribution was not demonstrated.


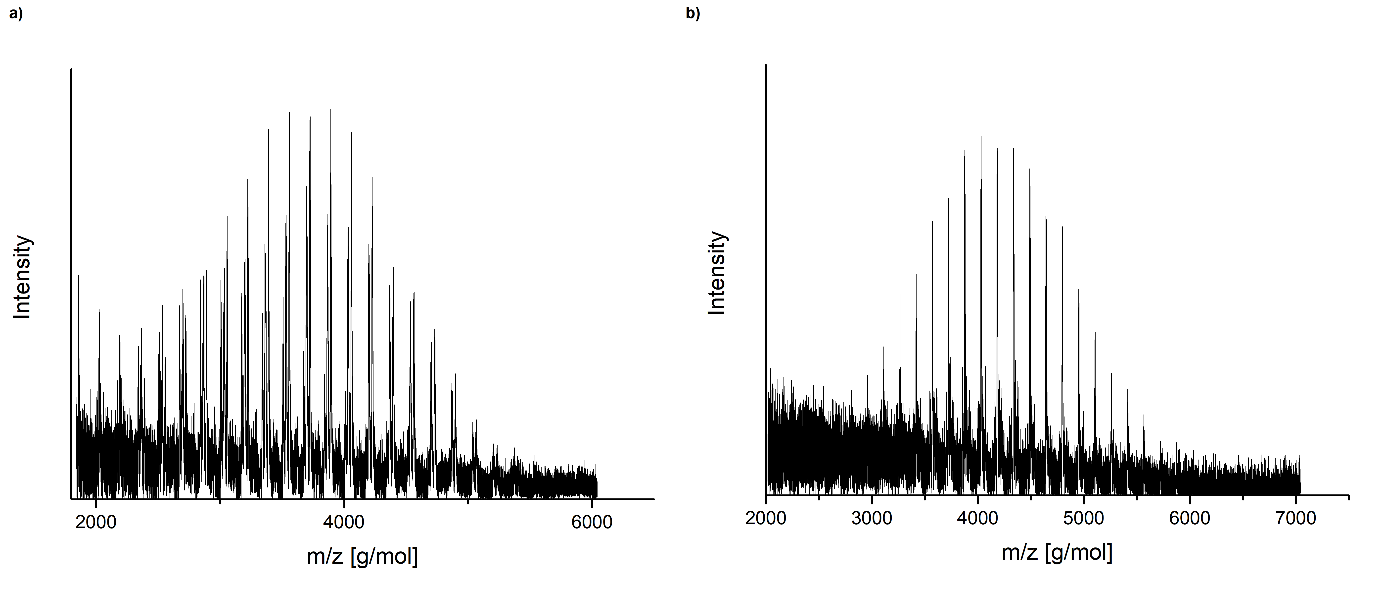


**Supporting Information Figure 9.** MALDI-TOF spectrum of a) P2Th2Ox_25_ b) P3Th2Ox_25_.

## Polymer characterisation

**Supporting Information Table 3.** Number average molecular weight (M_n_), polydispersity (Ð), glass transition temperature (T_g_) and yield (%) of synthesised polymers. a) theoretical value, b) calculated by H^1^-NMR, c) obtained from GPC (*T* = 40 °C, flow rate of 0.7 mL/min in hexafluoroisopropanol, calibrated using poly(ethylene glycol) standards), d) obtained from GPC (M_w_/M_n_), * became insoluble during polymerisation, e) NMR value not calculated as no reference signal identifiable, ** manually calculated by NETZSCH Proteus^©^

|  | **M_n_ [kg/mol]^a^** | **M_n_ [kg/mol]^b^** | **M_n_ [kg/mol]^c^** | **Ð^d^** | **T_g_ (°C)^**^** | **Yield (%)** |
| --- | --- | --- | --- | --- | --- | --- |
| **P2Th2Ox_25_^*^** | 4.00 | - | 1.10 | 1.19 | 95 | - |
| **P2Th2Ox_50_^*^** | 7.80 | - | 1.70 | 1.18 | 102 | - |
| **P3Th2Ox_25_** | 4.00 | - | 1.50 | 1.14 | 101 | - |
| **P3Th2Ox_50_** | 7.80 | - | 1.40 | 1.13 | 105 | - |
| **P2Th2Ozi_25_** | 4.30 | 2.80 | 7.50 | 1.27 | 55 | 77 |
| **P2Th2Ozi_50_** | 8.50 | 6.20 | 9.70 | 1.21 | 58 | 73 |
| **P3Th2Ozi_25_** | 4.30 | 4.00 | 2.90 | 1.09 | 59 | 84 |
| **P3Th2Ozi_50_** | 8.50 | 8.30 | 6.70 | 1.11 | 62 | 82 |
| **P2At2Ox_25_** | 4.30 | 3.20 | 1.20 | 1.39 | 59 | 40 |
| **P2At2Ox_50_** | 8.50 | 6.30 | 3.90 | 1.67 | 61 | 45 |
| **P3At2Ox_25_** | 4.30 | 3.00 | 3.10 | 1.12 | 59 | 63 |
| **P3At2Ox_50_** | 8.50 | 7.20 | 3.30 | 1.21 | 65 | 60 |
| **P2At2Ozi_25_** | 4.70 | 2.70 | 5.80 | 1.47 | 40 | 42 |
| **P2At2Ozi_50_** | 9.30 | 3.80 | 7.40 | 1.45 | 42 | 48 |
| **P3At2Ozi_25_** | 4.70 | 2.10 | 5.90 | 1.36 | 42 | 44 |
| **P3At2Ozi_50_** | 9.30 | 4.60 | 8.60 | 1.15 | 44 | 65 |
| **P(3Th2Ozi_20_-co-nrPOzi_5_)** | 3.98 | -^e^ | 3.50 | 1.28 | 51 | 83 |
| **P(3Th2Ozi_20_-co-nrPOzi_10_)** | 4.61 | -^e^ | 5.6 | 1.34 | 45 | 86 |
| **P(3Th2Ozi_20_-co-nrPOzi_20_)** | 5.88 | -^e^ | 7.8 | 1.29 | 38 | 75 |

## Solubility of thiophene-bearing molecules

Solubility was assessed by attempting to dissolve 1 mg in 1 mL of solvent and subsequently grouped into one of three groups.

**Supporting Information Table 4.** Solubility overview of monomers and polymers. Soluble (L) was denoted when product was able to be fully dissolved, L* denotes molecule is poorly soluble, X is insoluble (when less than 0.1 mg was dissolvable) and S indicates swelling when partial gelation was observed. S* indicates swelling is temperature dependent.

| **Solvent** | **Monomers** | **P(ThOx)** | **P(ThOzi)** | **P(AtOx)** | **P(AtOzi)** |
| --- | --- | --- | --- | --- | --- |
| **H_2_O** | X | X | S | - | S |
| **Acetone** | X | X | S* | X | - |
| **Ether** | L | X | X | X | X |
| **Chloroform** | L* | X | L | L | L |
| **Dichloromethane** | L* | X | L | - | - |
| **Acetonitrile** | L | X | L | L* | L |
| **Toluene** | L | X | L* | - | - |
| **Benzonitrile** | L | X | L | L | L |
| **Hexafluoroisopropanol** | L | L | L | L | L |
| **Ethanol/methanol** | X | X | S* | - | - |
| **Dimethyl sulfoxide** | - | X | L | L | L |
